# Beyond en-bloc turning: dynamics of head-pelvis coordination in 360° turns in people with Parkinson’s

**DOI:** 10.1186/s12984-026-01996-7

**Published:** 2026-05-20

**Authors:** Phaedra Leveridge, Yuri Russo, Genevieve K.R. Williams, Jiaxi Ye, Zijing Wang, Sarah E. Lamb, William R. Young

**Affiliations:** Department of Public Health and Sport Sciences, University of Exeter, Exeter, UK

**Keywords:** Turning, Parkinson’s disease, Freezing of gait, Segmental coordination, Vector coding

## Abstract

People with Parkinson’s Disease (PD) and Freezing of Gait (FOG) reportedly turn using an ‘en-bloc’ strategy, where the head and pelvis rotate together, unlike the head-leading movement seen in healthy adults. However, previous research relies on discrete maximum separation angles in 180° walking turns, despite recommendations to use 360° on-the-spot turns to better induce FOG. Moreover, existing studies do not capture the time-varying coordination of body segments throughout the turn. Our study aimed to investigate head-pelvis during 360° on-the-spot turns in people with PD and FOG (PD + FOG), PD without FOG (PD-FOG), and healthy controls (HC). Fifteen PD + FOG, fourteen PD − FOG (tested ON medication), and seventeen healthy controls (HC) completed repeated 360° turns while kinematic data were collected using marker-based motion capture. Head and pelvis angles were calculated across strides in the first, middle, and final sections of the turn. Coordination was quantified using vector coding, alongside discrete measures of angular difference and turning performance. Head–pelvis angular difference did not differ between groups. However, PD + FOG showed increased coordination variability compared to HC (4.9°, *p* = 0.005) and PD − FOG (3.5°, *p* = 0.047); an observation no longer significant after adjusting for Motor Section (III) of the Movement Disorder Society–Unified PD Rating Scale (MDS-UPDRS) motor scores (*p* = 0.249) and Mini Balance Evaluation Systems Test (MiniBEST) score (*p* = 0.051). PD + FOG also took more steps than PD − FOG (34.0, *p* = 0.008) and HC (6.5, *p* < 0.001), even after adjusting for covariates (MDS-UPDRS: *p* = 0.037; MiniBEST: *p* = 0.003). These findings suggest that people with PD do not necessarily turn more en-bloc than healthy controls. While head-pelvis coordination variability is higher in PD + FOG, this does not seem to be linked to FOG pathology per se, but rather balance deficits associated with disease severity. The impact of balance training on coordination variability and turn performance should be evaluated. Increased step count seems to be related to FOG, which could be a factor that might provoke FOG, but may serve as a compensatory strategy to promote postural stability. Future work should investigate how stride length/frequency modulation during turning affects postural stability and FOG occurrence.

**Supplementary Information:**

The online version contains supplementary material available at 10.1186/s12984-026-01996-7.

## Introduction

Turning, while standing or walking, is an essential component of human locomotion. Approximately one third of steps during daily living incorporate turns [1]. Typically, healthy adults first rotate their head in the direction of the turn, followed by their shoulders and pelvis [2, 3]. Head-pelvis coordination in turning appears to be altered in people with PD, with a more ‘en-bloc’ (in-line) strategy. Initiation of head rotation is postponed, resulting in a more closely coupled start of head and pelvis rotation in comparison with the top-down coordination in healthy controls [4]. Furthermore, maximum head-pelvis separation appears to be lower in people with PD compared to healthy older adults during 180° walking turns [5, 6]. Freezing of gait (FOG) is experienced by over 50% of people who have PD [7]. FOG is defined as a “brief, episodic absence, or marked reduction of forward progression of the feet despite the intention to walk”8 and is often experienced during turning [9]. People with PD and FOG take longer and more steps to turn than those without, with the difference between groups increasing with turning angle [10]. Spildooren and co-workers [5] found that head-pelvis angular difference in the first 65° of a 180° turn was lower in people with FOG compared to people with PD without FOG. A head-first strategy supports the visuomotor anticipation required to turn, as it provides a navigation function by giving a stable frame of reference [11]. A more closely coupled head and pelvis may therefore contribute to FOG, and may also reflect a reduced ability to coordinate segmental reorientation during turning. The so-called ‘Ziegler protocol’ [12], and paradigms using 360° standing turns, are increasingly used to elicit FOG in clinical and research settings [13–15]. While it is often suggested that FOG could be caused by a delay in head-pelvis separation [14, 16], previous research investigating head-pelvis coordination in people with FOG has focused on walking turns of 180° [5, 17]. A 360° turn places sustained demands on segmental reorientation and weight shifting, which may exacerbate FOG-related coordination deficits. Investigating head-pelvis coordination in 360° turns may provide a clearer understanding of how segmental coupling is impacted in people with PD and FOG, particularly as this task is commonly used to elicit FOG in experimental settings.

Interpretation of head-pelvis coordination during turning is complicated by the influence of disease severity on turning behaviour in PD. Turning step count, velocity and turn duration have all been associated with disease severity in people with PD [18, 19]. It remains unclear whether head-pelvis coordination is related to disease severity. FOG more frequently presents in people with moderate to severe PD, affecting more than 60% of those with a disease duration over 10 years [20]. Previous reports [21] have highlighted that disease severity should be statistically controlled for to isolate the contributions of FOG to behaviour. Previous comparisons of step count, duration, and head–pelvis angular differences between people with PD with and without FOG have not accounted for disease severity scores as a covariate [10]. By controlling for disease severity, we can determine whether observed deficits are inherent to FOG or reflect broader motor impairments associated with advanced PD. Identifying the most meaningful aspects of head-pelvis coordination to quantify can be challenging. Previous studies have used rotation onset times [6, 22–26], maximum head-pelvis angular difference [5, 6], and angular velocity [27, 28]. Since the demands on head-pelvis coordination change throughout a turn, different sections may pose unique motor control challenges. Spildooren and co-workers [5] captured angular difference at every 5° of pelvis rotation, providing a view of head-pelvis separation throughout the turn. However, focusing on angular differences limits understanding of how the head and the pelvis move together. For example, it is possible to have a large angular difference yet experience rigid en-bloc motion (i.e. after the initial decoupling the relationship among the body segments does not change). Vector coding is used to quantify relative movement and coupling between segments, describing the relative predominance of motion in one segment or another [29–32]. This analytical technique allows quantification of the proportion of motion occurring in-line, or in-phase, which reflects a rigid en-bloc movement pattern. To capture these dynamics across the whole turn, we can analyse head-pelvis coordination across each stride. Expressing coordination relative to the stride allows comparison across turns of different lengths. This approach ensures that head-pelvis dynamics are considered throughout the movement, rather than focusing on discrete points. Therefore, using vector coding in this context could provide a novel informative measure representing the dynamic relationship between the head and pelvis across each stride within the turn.

Coordinated movement requires the complex organisation of multiple degrees of freedom to perform actions [33]. Therefore, variability in joint coordination is typically seen in healthy individuals, allowing motor patterns to be stable and repeatable, yet giving flexibility to adapt to task constraints [34, 35]. In straight-line walking, head-pelvis coordination variability in the transverse plane is lower in people with PD than in healthy controls [36]. Similarly, people with PD have reduced gait adaptability (ability to adjust gait to target/obstacles) [37], and increased axial rigidity [38] which may hinder an ability to adapt to the dynamic requirements of turning. Nonetheless, head-pelvis coordination variability has not yet been investigated during turning in PD.

Using marker-based motion capture and analytic approaches novel in the context of turning in Parkinson’s disease (vector coding and continuous analysis), we investigated head–pelvis coupling and coordination variability during 360° turning. We aimed to determine whether en-bloc control during turning is a characteristic feature of people with Parkinson’s disease and freezing of gait (PD + FOG), or whether it is instead associated with disease severity or balance ability. This study compared head-pelvis coordination in 360° on-the-spot turns in people with PD and FOG (PD + FOG), PD without FOG (PD-FOG), and healthy controls (HC). It was hypothesised that: (i) head-pelvis angular difference would be lower, and the percentage of the stride in-phase would be higher in PD + FOG than HC and PD-FOG; (ii) head-pelvis coordination variability would be lower in PD + FOG than PD-FOG and HC (iii) increased disease severity, and poorer balance ability, would exacerbate differences between PD + FOG and PD-FOG across head-pelvis coordination and turning performance metrics (turn duration, and number of steps).

## Methods

### Study design

This study followed an observational cross-sectional design. All participants with PD were tested ON medication – (approximately 60 min after their last dose) of dopaminergic medication. If required, participants were allowed to re-take their medication to ensure they were ON medication. The study was approved by the local institutional review board of the University of Exeter (21-12-08-B-02, Department of Public Health & Sport Sciences).

### Participants

Forty-two people with PD were recruited through local Parkinson’s UK branches. Twenty-three self-reported frequently experiencing FOG (PD + FOG), and nineteen did not experience FOG (PD-FOG). Participants in each group required a diagnosis of idiopathic PD using the UK Brain Bank Criteria [39]. Participants were included in the PD + FOG group if they self-reported FOG according to the first item of the New Freezing of Gait Questionnaire (NFOG-Q) [40]. Twenty-four older adults (aged 55 years+) were recruited as controls (HC). Participants were required to be able to walk unsupported (> 1 min), have no cognitive impairment (Montreal Cognitive Assessment (MOCA) [41], Score > 20), have no comorbidities that would affect balance or walking, and be aged 55 + years old. All participants provided written informed consent prior to participating in the study.

### Procedure

**Fig. 1:**
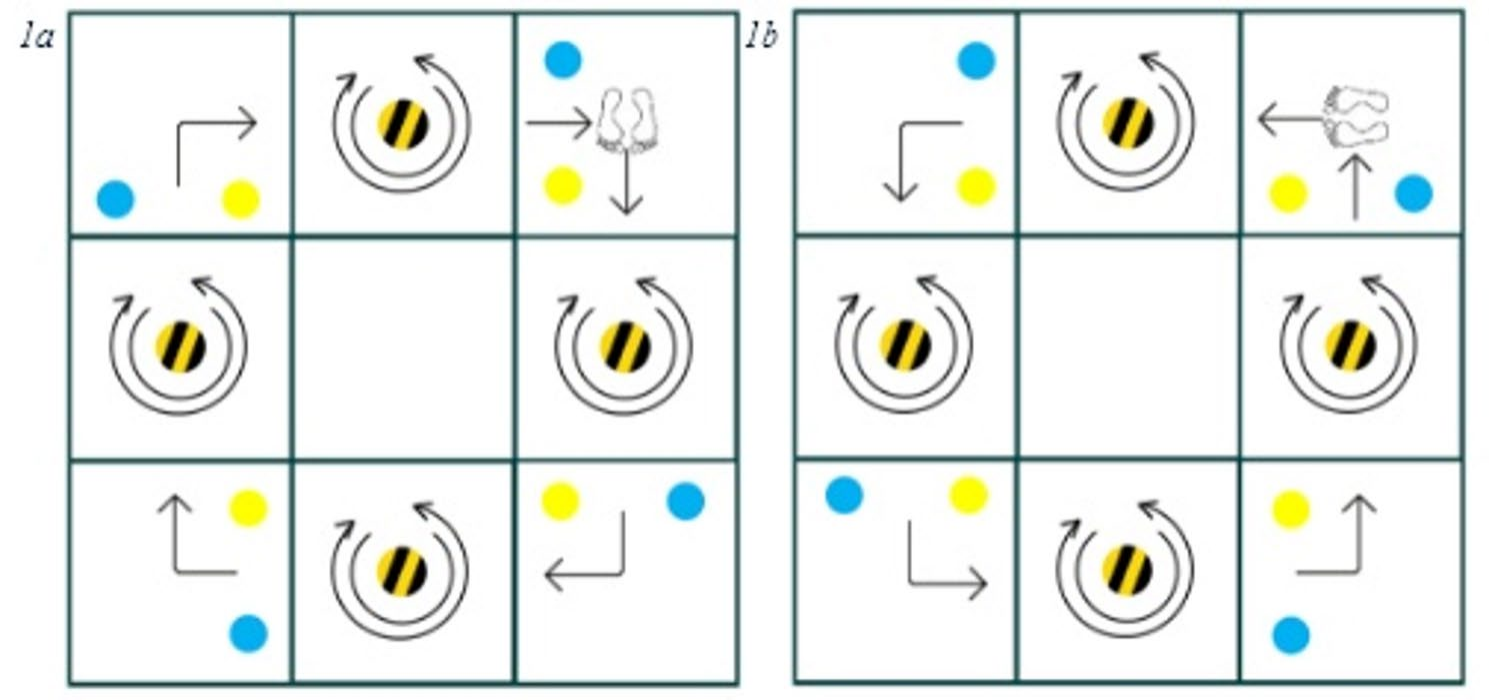
–1a depicts the walking and turning task in a clockwise direction, and 1b in an anticlockwise direction. Yellow and black striped circles depict the target where participants completed 360° turns in each direction. Yellow and blue circles show the location of the cones. The footprint denotes the start point. Arrows show direction of travel

After providing informed consent, participants with PD were rated by a trained examiner (YR or WY) on the Motor Section (III) of the Movement Disorder Society-Unified PD Rating Scale (MDS-UPDRS) [42]. Mini Balance Evaluation Systems Test (MiniBEST) [43] and the MOCA [41] were administered to all participants. All participants were asked to complete a walking task (shown in Fig. 1) in a clockwise direction, followed by an anti-clockwise direction. They were asked to walk through cones to a yellow and black striped circular target in the capture area. Then, they were instructed to stop on the target and complete a modified Ziegler protocol [12] - where a 360˚ turn was performed in one direction, then a second 360˚ turn was performed in the opposite direction. Participants were asked to stop before and after each turn to ensure reliable attribution of steps to individual turns and turn phases. Once the turning task had been completed, the participant continued through the cones to the next target and repeated the task. Participants were instructed when to change from travelling in the clockwise direction to anticlockwise, completing around 6 targets in each direction.

Kinematic data were recorded during the task using a 19-camera motion capture system (Prime 13, Optitrack, USA) with a sampling rate of 100 Hz. Thirty-nine retroreflective markers were placed on each participant’s skin and clothing according to the full-body Plug-in Gait model [44]. If clothing was not skin-tight, or if preferred, participants wore an Optitrack Motion Capture Suit where markers were attached using Velcro.

### Data analysis

Data were processed in Visual3D (C-Motion, Inc., Germantown, MD, USA), and further processed in MATLAB (Version R2024a, MathWorks Inc., USA) using custom scripts and functions. Data were filtered using a low pass 4th order Butterworth filter with a cut-off frequency of 6Hz [45]. The head and pelvis were defined as rigid bodies, and transverse plane head and pelvis angle in relation to the global coordinate system was extracted, where a pelvis angle of 0° corresponded to the position at the start of the turn. The start of each turn was defined as when participants stopped in quiet stance on the target prior to each 360° turn, or the point where the head reached 5° rotation (whichever point was earlier). Turns were excluded from analysis if there was no clear stop before, after, or in between turns, to ensure reliable attribution of steps to individual turns and turn phases. Video recordings were used to identify turns to be excluded from analysis due to the presence of FOG events, assessed through the annotation of video recordings by a panel of trained researchers, as FOG alters turning behaviour [5, 46] and would prevent valid comparisons across groups. Participants were excluded from the analysis if they were unable to complete any 360° turns without FOG, and if they had any turning problems unrelated to PD.

**Table 1:** This table summarises the rationale for inclusion of the outcome variables examined in this study

	Variable	Hypothesis	Justification
Discrete	Turn duration (s)	(iii)	Turn duration was included as a summary measure of turning performance to contextualise coordination outcomes and to assess whether increasing disease severity is associated with slower turning.
	Number of steps	(iii)	The total number of steps provides a marker of turning performance to provide context for coordination analysis results. To understand the influence of disease severity on turning performance.
	Maximum angular difference (°)	(i) (iii)	Maximum head–pelvis angular difference was included as a discrete measure of peak segmental decoupling during turning. As this metric has been widely used to characterise en-bloc turning in PD, its inclusion allows direct comparison with existing literature.
	Average angular difference (°)	(i) (iii)	Average angular difference summarises overall head-pelvis separation throughout the turn, providing a global measure of segmental coordination.
	Percentage in-phase (%)	(i) (iii)	The percentage of the stride in-phase quantifies the proportion of time the head and pelvis are moving together in the same direction, reflecting rigid en-bloc motion.
	Coordination variability (°)	(ii) (iii)	Mean coordination variability (circular standard deviation of the coupling angle across strides in each part of the turn) provides a single summary metric of movement variability, enabling comparison of overall variability between groups
Continuous	Angular difference (°)	(i) (iii)	Continuous head–pelvis angular difference, normalised to 100% of the turn and each stride, was analysed to characterise how segmental separation evolves throughout the turn. This approach allows identification of when coordination differences emerge, supporting detailed evaluation of dynamic segmental control across groups.
	Coordination variability (°)	(ii) (iii)	Coordination variability (circular standard deviation of coupling angle in strides in each part of the turn) allows us to understand the variation and adaptability of participants in each section of the turn. This allows detailed analysis of when and how coordination deficits emerge to understand dynamic segmental control.

Maximum head-pelvis angular difference was the maximum difference across the whole turn, and average angular difference was the average difference across the whole turn. Head-pelvis angular difference and coupling angle were calculated across the stride from heel strike to heel strike of the foot contralateral to the direction of turn normalised from 0 to 100% (as described in the supplementary materials). Strides were categorised into three sections of the turn—start, middle, and end [47]—based on the pelvis rotation angle (0–120°, 120–240°, and 240–360°, respectively) during 60% of the stride. Data preceding the first full stride and following the last full stride were excluded from analysis.

**Fig. 2:**
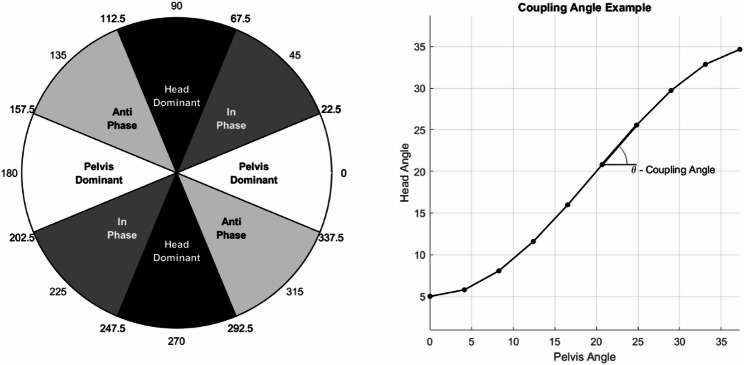
Vector coding analysis procedures. Coupling angle calculated from the relative motion data as the angle from the right horizontal (right). This coupling angle provides the basis for binning the phase relations into eight distinct coordinative patterns with a range of ± 22.5° (left). Final analysis was performed on the ‘in-phase’ bins, where head and pelvis are moving in the same direction

Vector coding coupling angle was calculated across each stride based on head (H) and pelvis (P) rotation angle time series according to the following Eq. (1):

1 $$\:{\theta\:}_{i}={\mathrm{tan}}^{-1}(\frac{{P}_{(i+1)}-{P}_{\left(i\right)}}{{H}_{(i+1)}-{H}_{\left(i\right)}})$$

where 0° ≤ θ ≤ 360° and *i* represents consecutive data points [30, 31]. Coupling angle is computed between each pair of successive data points ($\:i,\:i+1$) of head and pelvis angles as a time series. The resulting series of $\:{\theta\:}_{i}$ values form the continuous variable (as in Fig. 2, right), where the interpretation of the coupling angle is depicted in Fig. 2, left. The percentage of each stride where the coupling angle was in the in-phase coordination bin (22.5–67.5° and 202.5-247.5°) was calculated [29] (as described in Fig. 2, left). Coordination variability was calculated as circular standard deviation of coupling angle for the start, middle, and end of the turn for each participant [48]. A discrete measure of coordination variability was defined as the mean of the circular standard deviation across the stride.

### Statistical analysis

Discrete statistical analysis was performed in SPSS (Version 28, IBM, Chicago, IL). A two-way mixed model ANOVA (3 × 3) was used to examine differences between groups (PD + FOG, PD-FOG and HC) and part of the turn (start, middle, and end) in the percentage of the stride in-phase, and coordination variability. A one-way ANOVA was used to investigate differences between groups in maximum/average angular difference, turn duration, and number of steps. A mixed-model ANCOVA, and one-way ANCOVA were performed using MDS-UPDRS (FOG item removed (3.11)) as a covariate to account for differing disease severity between PD + FOG and PD-FOG groups. Another mixed-model ANCOVA, and one-way ANCOVA were performed using MiniBEST score as a covariate to determine the contribution of balance ability to turning behaviour. Data were tested for sphericity using Mauchly’s test, and Greenhouse-Geisser corrections were applied if violated. Data presented as mean ± standard deviation (SD), and standard error (SEM) where values have been adjusted for covariate. SEM has been presented to reflect precision of the adjusted estimates and facilitates between-group comparisons by showing uncertainty in the mean rather than individual variability. Pearson’s correlations were carried out between coordination variability, turn duration, and MDS-UPDRS/MiniBEST scores across all groups to determine how closely coordination variability, turn performance and disease severity/balance ability are linked.

Using the SPM1D package (v.0.4.2) [49, 50], statistical parametric mapping (SPM) two-way ANOVA with repeated-measures on one factor was used to compare angular difference and coordination variability across the stride between groups and part of the turn. To account for MDS-UPDRS and MiniBEST scores, an SPM ANCOVA was used. Since the SPM1D package does not directly implement ANCOVA’s, an SPM regression was run and a two-way ANOVA with repeated-measures on one factor used to compare residuals between groups and part of the turn. Bonferroni corrections were applied to post-hoc comparisons. For all tests, the level of significance was set at α = 0.05.

## Results

### Participant demographics

Fifteen PD + FOG, fourteen PD-FOG and seventeen HC were included in these analyses. Eight PD + FOG, five PD-FOG and two HC were excluded (see supplementary materials for further details). Participants in each group were well matched in demographic characteristics, and MOCA scores (Table 2). Balance abilities (MiniBEST) were better in HC than PD + FOG (*p* = 0.043) and PD-FOG (*p* < 0.001), and were worse in PD + FOG than in PD-FOG (*p* = 0.018). Disease severity was worse in the PD + FOG group than PD-FOG (both Hoehn and Yahr (H&Y) stage, and MDS-UPDRS score).

**Table 2:**
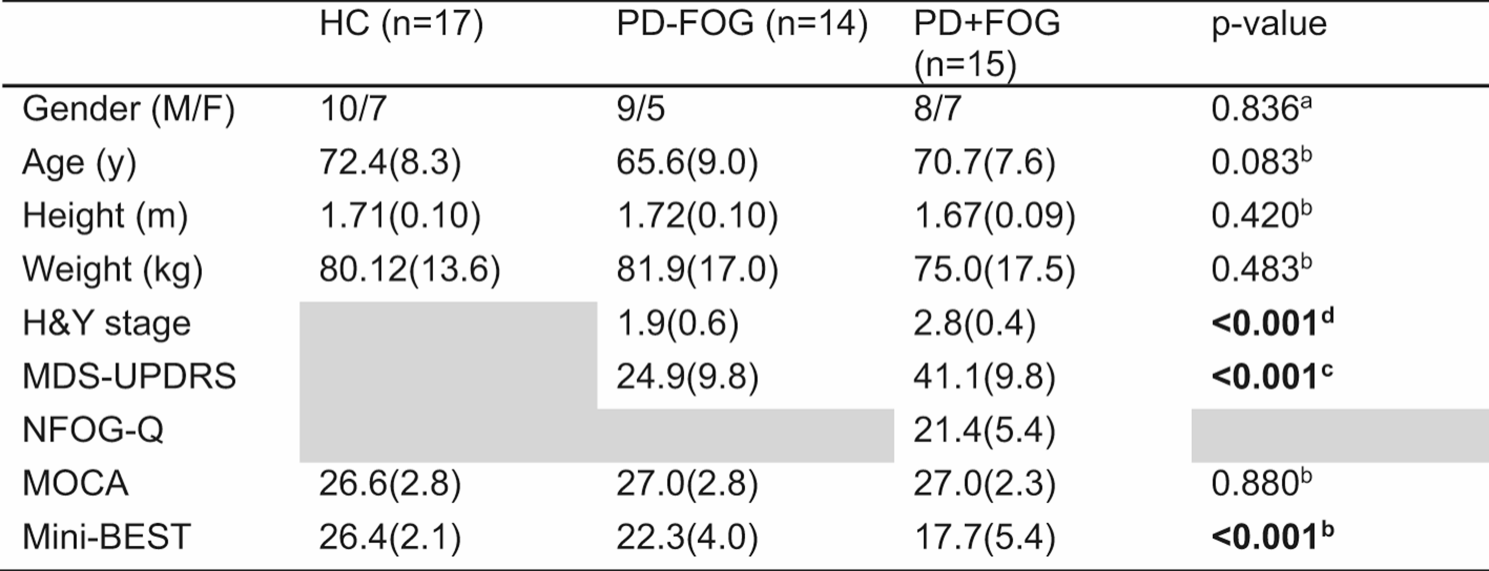
Demographics and clinical characteristics of participants. HC, PD-FOG and PD + FOG

Significant differences (*p* < 0.05) are shown in bold. Grey shading indicates values are not applicable. (a) Gender distribution was assessed with Chi-square test of independence. (b) p-values of one-way ANOVA. (c) p-value of independent t-tests. (d) p-value of Mann-Whitney U test

### Turning performance

The total number of steps per turn was significantly higher in PD + FOG than PD-FOG and HC (see Table 3). These differences remained in both covariate analyses: adjusting for MDS-UPDRS, as well as adjusting for MiniBEST. PD + FOG took longer to turn than HC. However, when including MiniBEST score as a covariate, while a main effect of group remained, there was no longer a difference between PD + FOG and HC when Bonferroni corrections were applied (*p* = 0.061).

**Table 3:**
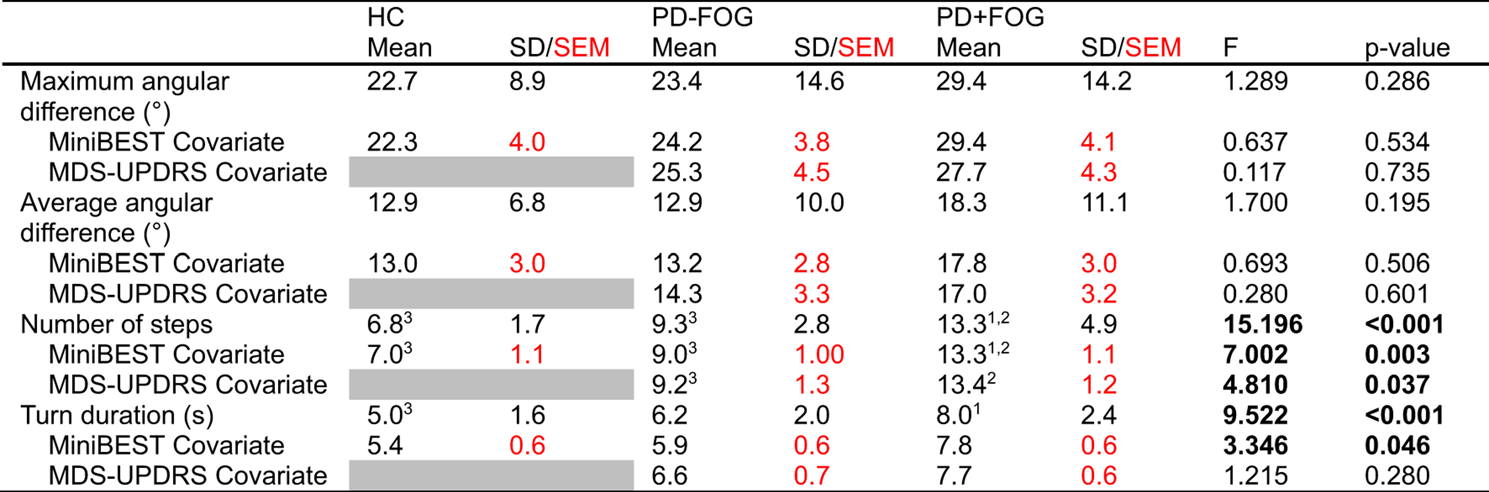
Turning characteristics (mean and SD/SEM in red) in HC, PD-FOG and PD-FOG

Results of the one-way ANOVA and one-way ANCOVA (covariate shown in the left column). Significant main effects (*p* < 0.05) are shown in bold. Grey shading indicates values are not applicable. Significant post-hoc comparisons with Bonferroni corrections are shown in superscript: (1) Significantly different to HC, (2) Significantly different to PD-FOG, (3) Significantly different to PD + FOG

**Table 4:**
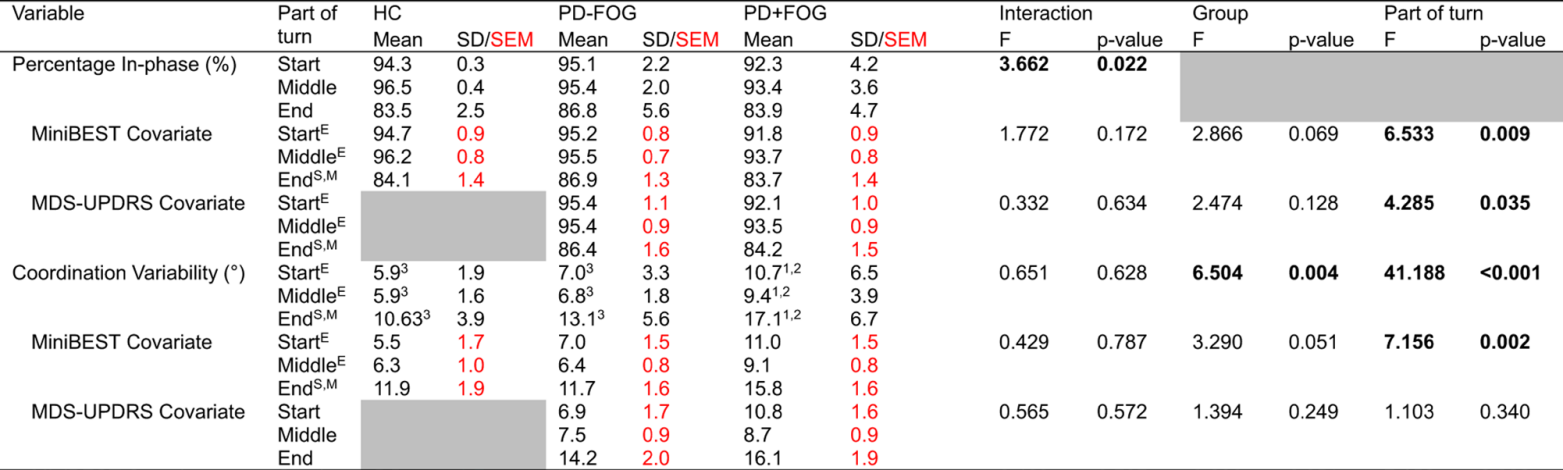
Turning characteristics (mean and SD/SEM in red) in HC, PD-FOG and PD-FOG in each part of the turn

Results of the mixed model ANOVA and mixed model ANCOVA (covariate shown in the left column). Significant main effects (*p* < 0.05) are shown in bold. Grey shading indicates values are not applicable. Significant post-hoc comparisons with Bonferroni corrections are shown in superscript: (1) Significantly different to HC, (2) Significantly different to PD-FOG, (3) Significantly different to PD + FOG; (S) Significantly different to the start, (M) Significantly different to the middle, (E) Significantly different to the end

### En-bloc turning - angular difference

There were no significant differences between groups in maximum or average angular difference (as shown in Table 3).

**Fig. 3:**
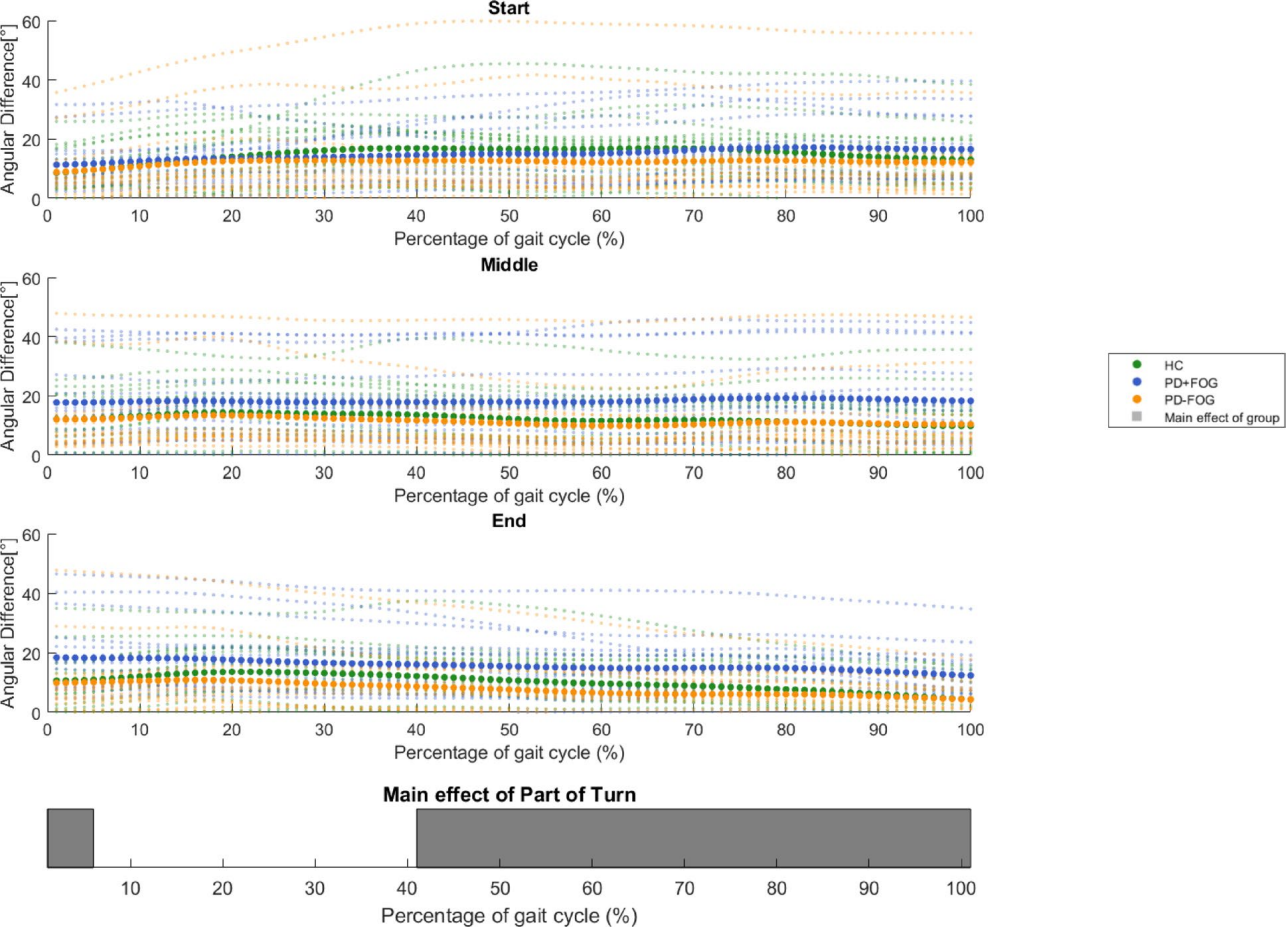
Head-pelvis angular difference across the stride at the start, middle and end of the turn in HC (green), PD + FOG (blue) and PD-FOG (orange) groups. Transparent data points represent angular difference for each participant, and bold points represent the mean for each group. There was no significant main effect of group. Significant main effects of part of turn are presented in the bottom plot

Based on SPM analysis of head-pelvis angular difference across the stride in each third of the turn, there was no significant interaction effect or main effect of group (as shown in Fig. 3). Angular difference was significantly lower from 54 to 100% of the stride in the end part of the turn compared to the start part of the turn. Between 42 and 100% of the stride cycle, the angular difference was lower for strides occurring at the end of the turn than for strides in the middle of the turn.

### En-bloc turning – percentage of the stride inphase

There was a significant interaction between group and part of the turn in percentage of the stride in the in-phase coordination bin (*p* = 0.022, shown in Table 4). In the start of the turn, there was significantly less of the stride in the in-phase bin in the PD + FOG group (92.3 ± 4.1%) compared to the PD-FOG group (95.1 ± 2.2%; *p* = 0.019). In the middle of the turn there was less of the stride in the in-phase bin in the PD + FOG group (93.4 ± 3.6%) compared to the HC group (96.5 ± 0.4%; *p* = 0.002). In the HC group, start (94.3 ± 0.3%), middle, and end (83.5 ± 2.5%) were significantly different (*p* < 0.001 across all). In PD + FOG and PD-FOG groups, the start and middle (PD-FOG: 95.4 ± 2.0%) were different to the end of the turn (PD + FOG: 83.9 ± 4.7%; PD-FOG: 86.8 ± 5.6%; *p* < 0.001 for all comparisons).

When including either MiniBEST score and MDS-UPDRS score as a covariate, there was no longer an interaction effect, nor was there a main group effect. When accounting for MDS-UPDRS score, the end of the turn (85.3 ± 1.0%) had a significantly lower percentage in-phase than the start (93.7 ± 0.6%, *p* < 0.001) and middle of the turn (94.4 ± 0.6%, *p* < 0.001). When accounting for MiniBEST score, the end of the turn (84.9 ± 0.7%) had a lower percentage of the stride in-phase compared to the start (93.9 ± 0.4%, *p* < 0.001) and middle (95.1 ± 0.7%, *p* < 0.001). Here, the start of the turn had less of the stride in-phase compared to the middle of the turn (*p* < 0.001).

### Coordination variability

There was no interaction effect between group and turn section in discrete coordination variability (Table 4). PD + FOG (12.4 ± 1.0°) had higher coordination variability than PD-FOG (8.9 ± 1.0°, *p* = 0.047) and HC (7.5 ± 1.1°, *p* = 0.005). Coordination variability was higher at the end of the turn than at the start (*p* < 0.001) and middle (*p* < 0.001). When including either MDS-UPDRS and MiniBEST score as a covariate, there was no longer a main effect of group. The main effect of part of turn remained when accounting for MiniBEST score (*p* = 0.002), but not MDS-UPDRS (*p* = 0.340).

Coordination variability across all groups was associated with the turn duration and balance ability. Coordination variability was not correlated with disease severity in PD + FOG and PD-FOG (*p* > 0.05). There was a moderate positive correlation between turn duration and coordination variability at the start (*r* = 0.393, *p* = 0.015) and end of the turn (*r* = 0.387, *p* = 0.008), and a strong positive correlation in the middle (*r* = 0.693, *p* < 0.001). MDS-UPDRS scores showed no significant correlation with coordination variability (*p* > 0.05). MiniBEST scores had a moderate negative correlation with coordination variability in the middle (*r*=-0.341, *p* = 0.027) and end of the turn (*r*=-0.406, *p* = 0.008). MDS-UPDRS scores showed a moderate positive correlation with turn duration (*r* = 0.390, *p* = 0.036), while MiniBEST scores had a moderate negative correlation (*r*=-0.445, *p* = 0.003).

**Fig. 4:**
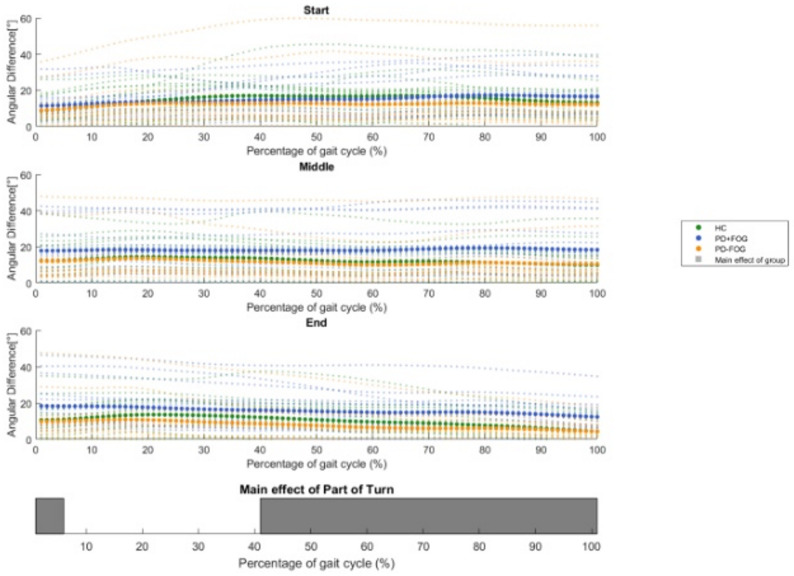
Head-pelvis coordination variability across the stride at the start, middle and end of the turn in HC (green), PD + FOG (blue) and PD-FOG (orange) groups. Transparent data points represent coordination variability for each participant, and bold points represent the mean for each group. There was no significant main effect of group when accounting for MDS-UPDRS (or MiniBEST) score. Significant main effects of part of turn when accounting for MDS-UPDRS score are presented in the bottom plot (main effect of part of turn is the same when accounting for MiniBEST score)

In SPM analysis of coordination variability across the stride in each third of the turn, there was no significant interaction effect between group and part of turn. The PD + FOG group had significantly higher coordination variability than the PD-FOG group between 37 and 41% and 53–59% of the stride. The PD + FOG group had higher coordination variability at 30%, 36–38%, 40–41%, 43–55% of the stride. There were no significant differences in coordination variability between PD-FOG and HC groups (*p*>0.05). In SPM analysis of the residuals from the regression between MDS-UPDRS/MiniBEST, and coordination variability (SPM ANCOVA), no group differences were observed (as shown in Fig. 4). There was no significant difference between strides in the start and middle of the turn. Between 56% and 100% of the stride cycle, the coordination variability was higher for strides occurring at the end of the turn than for strides in the start of the turn. Strides in the middle of the turn had lower head-pelvis coordination variability from 47 to 48%, 52–54% and 56–100% of the stride cycle than in strides occurring at the end of the turn.

## Discussion

This study aimed to investigate head-pelvis coordination in 360° on-the-spot turns in people with PD and FOG, PD without FOG, and healthy controls. Contrary to our hypothesis, results showed no differences between PD + FOG, PD-FOG and HC groups in angular difference across the stride, or in maximum angular difference during the turn. There were also no differences between PD + FOG, PD-FOG and HC groups in the percentage of the stride in which the head and pelvis were in-phase. Contrary to our second hypothesis, coordination variability was higher in PD + FOG than PD-FOG and HC, but this effect was no longer significant after accounting for disease severity and balance ability. PD + FOG took more steps to complete the turn than both PD-FOG and HC, even when controlling for these covariates.

To quantify head-pelvis coordination during turning, we applied both previously used definitions of head-pelvis coordination (angular difference across the stride and maximum angular difference) as well as vector coding techniques that overcome limitations of focusing on maximum difference. Despite applying both methods, we found no evidence of more en-bloc turning in PD + FOG, or PD-FOG compared to HC. Spildooren and colleagues [5], who tested 13 individuals with PD + FOG, 14 with PD − FOG, and 14 age-matched controls, found similar maximum head–pelvis angular differences in 180° walking turns in people with PD (without FOG = 27.3°; with FOG = 25.7°) to the present study (PD − FOG = 23.4 ± 14.6°; PD + FOG = 29.4 ± 14.2°). However, their control group showed greater maximum head–pelvis differences (35.4°) than our HC group (22.8 ± 8.9°). The typical head-first strategy deployed by healthy older adults is disrupted when turning on-the-spot towards predictable targets [51, 27, 52, 53]. A smaller angular difference may reflect prioritisation of stability over visual input, as the target and movement path are known. Thus, the lower maximum angular difference in our HC group (compared to previous work [5]) may be due to the reduced requirement for a head-first strategy when turning on-the-spot to pre-determined targets, unlike in the 180° walking turn paradigm. En-bloc turning in people with PD and older adults seems to be task-dependent, but it is unclear to what extent the nature of the task affords the need to gather information (through visual input). To understand the most influential aspect of the task, coordination should be compared across standing and walking turns at different angles.

We found evidence of reduced in-phase coordination between the head and the pelvis in the strides at the end of the turn across all groups. Furthermore, SPM analysis revealed that angular difference was lower at the end of strides in the last part of the turn compared to the start and middle. This suggests that there is reorientation of the head and pelvis towards the end of the 360° turn. If the turn were completed in an en-bloc manner, head-pelvis angular difference and in-phase coordination would remain consistent throughout. We therefore found no evidence of en-bloc turning in any group. In previous literature, en-bloc turning is typically defined based on group-to-group comparisons of angular difference. For example, Yang et al. [6] found that, in 180° walking turns, the maximum head-pelvis angle was 18.41 ± 6.06° in people with PD, which was significantly lower than 22.77 ± 1.62° in a healthy control group; while significance was reached, interpretation of a more en-bloc strategy is ambiguous. Future work on en-bloc turning should analyse movement patterns throughout the turn to determine the presence of an en-bloc strategy, rather than relying on group comparisons.

We found higher head-pelvis coordination variability in PD + FOG compared to PD-FOG and HC, with significant correlations between coordination variability and turn duration (start: *r* = 0.393, middle: *r* = 0.693, end: *r* = 0.387), and between coordination variability and MiniBEST score (middle: *r*=-0.341; end: *r*=-0.387). These results do not align with the loss of complexity hypothesis [35, 54], but could instead be a phenomenon arising due to the relative difficulty posed by a given task. Specifically, exploratory behaviour may arise because the 360° turning task is designed to challenge motor control to elicit FOG. Furthermore, coordination variability was higher in the strides at the end of the turn across all groups. As a 360° turn is longer than most turns that occur in daily living [18], the end of the turn is more challenging, indicating that the task demands get progressively greater, exposing weaknesses. FOG occurs most frequently towards the end of turning (albeit in 180° turns) [5, 46], which may be due to this variability acting as a sequence effect [55] from the increasing challenge of movement control. Variability may accumulate and reach a threshold where FOG is triggered, explaining why 360° turning elicits FOG more than other turning tasks [14]. This finding parallels observations in motor learning paradigms where the stages of motor skill acquisition are associated with a U-shaped trajectory in coordination variability [56]. Here, coordination variability is highest in the least and most skilled performers, and lowest in intermediate-level performers [56]. This reflects exploratory behaviour in novices, but an ability to exploit degrees of freedom in the motor system in skilled performers. Exploratory behaviour seen in the 360° turn could reflect both lower levels of skill and cautious behaviour. For a given task, while we may see more en-bloc turning, head-pelvis coordination variability likely better reflects the underlying motor control. Future research should therefore focus on the relationship between variability and FOG.

Head-pelvis coordination variability seems to be more closely related to balance ability than disease severity. While coordination variability across strides at the middle and end of the turn was correlated with balance ability, there was no correlation with MDS-UPDRS score. Furthermore, when including MiniBEST score as a covariate, the group effect in discrete coordination variability, and, in SPM analysis, the group effect in the middle of the stride, was no longer significant. This reflects previous findings, as Cheng et al. [57] found that balance ability influenced turn duration across a 180° turn more than MDS-UPDRS score, or NFOGQ score.

Head-pelvis coordination variability does not seem to be directly linked to FOG pathology. When including MDS-UPDRS score as a covariate, there were no longer differences between PD + FOG and PD-FOG. FOG more frequently presents in people with moderate to severe PD [20], so it is important to control for disease severity to understand whether behaviour is inherently linked to FOG pathology or more general progression of motor symptoms. Previous research comparing people with PD with and without FOG typically shows no statistically significant differences in MDS-UPDRS or H&Y score between groups, often sampling to match disease severity [10, 17, 45, 46]. We believe that there are two main issues with this approach. First, selective sampling to match groups based on severity of motor symptoms compromises the generalisability of the group with FOG. Secondly, the absence of significant differences between groups cannot be unambiguously interpreted as evidence of no meaningful differences in disease severity, especially in highly heterogeneous samples where large variability in disease severity scores hinders the ability to detect between-group differences. We argue that statistically controlling for MDS-UPDRS score overcomes both issues, so should be considered for further work.

The number of steps taken to complete a 360° turn seems to be linked to FOG pathology. The PD + FOG group took more steps to turn compared to PD-FOG and HC, even when accounting for balance ability and disease severity. This aligns with previous results as, in a meta-analysis [10], people with PD and FOG took more steps in turns than those without FOG (mean difference of 4.98 steps in 360° turning). In straight line walking, inducing shorter-than-preferred step length increased the number of FOG events [55], and stride length progressively reduces prior to FOG [58]. An increased number of steps may therefore contribute to the onset of FOG. Alternatively, the increased number of steps could reflect prioritisation of stability. Shorter, more frequent steps represent a more stable strategy because the centre of mass (COM) is closer to the moving base of support [59]. A reduction in stride length has been found to increase margins of stability in the backwards direction (distance in anterior-posterior direction between COM and posterior border of the leading foot) during forward walking [60]. Furthermore, decreases in stride length have been found in anticipation of [61], and response to perturbations [62–64]. Smaller stride length is therefore likely to represent a strategy to increase mediolateral and backward margins of stability and thus prevent postural instability in the presence of perturbations (including unexpected FOG events). An increased number of steps in the turn may therefore be a strategy to prioritise stability over movement efficiency.

Given that head–pelvis coordination variability was more strongly associated with balance ability than with disease severity, future work could employ balance training to improve turning and evaluate the potential impact on coordination variability. As balance ability is modifiable, interventions targeting dynamic balance may influence the regulation of segmental coordination during turning. Since we found no clear evidence of en-bloc turning in the 360° turning task, interventions aimed solely at increasing head–pelvis angular separation might be less effective than general balance training focused on improving movement consistency. Furthermore, future work should investigate how stride length/frequency modulation during turning affects postural stability and FOG occurrence. Identifying the precursor behaviour to FOG events during turning would allow us to determine the contribution of variability and step behaviour to FOG.

There are several limitations in this study. Firstly, people with PD were only assessed while ‘ON’ medication. While this is more clinically meaningful, as most tasks and interventions (including turning) are performed ‘ON’ medication, medication status can influence turning behaviour for the better [17]. Inclusion to the PD + FOG group was based on self-report of FOG from the NFOGQ. FOG was observed in most participants, either during the task or on other occasions during the laboratory visit. However, formally recording the presence of ‘definite FOG’ during the visit for group allocation would be recommended for further work. Finally, strides were defined based on the contralateral foot heel strike, meaning dynamics of head-pelvis coordination were not considered before the first contralateral heel strike. Likewise, segment rotation onsets were not reported, as heterogeneity of methods of calculating onset in the literature makes values incomparable.

In conclusion, people with PD (PD + FOG and PD-FOG groups) did not turn more en-bloc in a 360° on-the-spot turn compared to healthy older adults (HC group). Head-pelvis coordination variability was higher in the PD + FOG group. While we may see more en-bloc turning in a task, we propose that head-pelvis coordination variability likely better reflects the underlying motor control, so should be the focus of future work. After correcting for disease severity (MDS-UPDRS), there were no differences between groups. Therefore, future research should account for disease severity when investigating characteristics of turning and gait in people with PD and FOG. People in the PD + FOG group took more steps to turn, even when controlling for disease severity. Although it remains unclear whether this increases the likelihood of FOG or is compensatory behaviour. Further research should investigate stepping behaviour and head-pelvis coordination leading to FOG episodes in 360° turning to understand the contribution of head-pelvis coordination to FOG.

## Electronic Supplementary Material

Below is the link to the electronic supplementary material.

Supplementary Material 1.

## Data Availability

The datasets supporting the conclusions of this article are available in the Open Science Framework repository, DOI 10.17605/OSF.IO/YDB4Z, [https://osf.io/ydb4z/].
