# Supplementary material for: Beyond en-bloc turning: dynamics of head-pelvis coordination in 360° turns in people with Parkinson’s
Source: J Neuroeng Rehabil. 2026 May 20;23:221. doi: 10.1186/s12984-026-01996-7 (PMC13371622; doi:10.1186/s12984-026-01996-7)
Supplement: Supplementary file 1 — Supplementary Material 1. [file 12984_2026_1996_MOESM1_ESM.pdf]

Supplementary Materials

1. Effect Sizes

Table 1 - Comparison of turning characteristics in HC, PD-FOG and PD-FOG in each part of the turn. Results of the mixed model ANOVA and mixed model ANCOVA (covariate shown in the left column). Significant main effects ($p < 0.05$) are shown in bold. Grey shading indicates values are not applicable. F statistic, p-value and effect size (η_p^2) are provided for each comparison. Significant main effects ($p < 0.05$) are shown in bold.

Variable	Interaction			Group			Part of turn		
	F	p-value	η_p^2	F	p-value	η_p^2	F	p-value	η_p^2
Percentage In-phase (%)	**3.662**	**0.022**	**0.146**						
MiniBEST Covariate	1.772	0.172	0.021	2.866	0.069	0.131	**6.533**	**0.009**	**0.147**
UPDRS Covariate	0.332	0.634	0.022	2.474	0.128	0.087	**4.285**	**0.035**	**0.141**
Coordination Variability (°)	0.651	0.628	0.036	**6.504**	**0.004**	**0.271**	**41.188**	**<0.001**	**0.541**
MiniBEST Covariate	0.429	0.787	0.027	3.290	0.051	0.175	**7.156**	**0.002**	**0.188**
UPDRS Covariate	0.565	0.572	0.039	1.394	0.249	0.055	1.103	0.340	0.044

Table 2 - Comparison of turning characteristics in HC, PD-FOG and PD-FOG. Results of the one-way ANOVA and one-way ANCOVA (covariate shown in the left column). Number of participants included in each comparison (n), F statistic, p-value, and effect size (η_p^2) are provided for each comparison. Grey shading indicates values are not applicable. Significant main effects ($p < 0.05$) are shown in bold.

	HC	PD-FOG	PD+FOG			
	n	n	n	F	p-value	η_p^2
Maximum angular difference (°)	17	14	15	1.289	0.286	0.057
MiniBEST Covariate	15	12	15	0.627	0.534	0.032
UPDRS Covariate		14	15	0.117	0.735	0.004
Average angular difference (°)	17	14	15	1.700	0.195	0.073
MiniBEST Covariate	15	12	15	0.693	0.506	0.035
UPDRS Covariate		14	15	0.280	0.601	0.011
Number of steps	17	14	15	**15.196**	**<0.001**	**0.414**
MiniBEST Covariate	15	12	15	**7.002**	**0.003**	**0.269**
UPDRS Covariate		14	15	**4.810**	**0.037**	**0.156**
Turn duration (s)	17	14	15	**9.522**	**<0.001**	**0.307**
MiniBEST Covariate	15	12	15	**3.346**	**0.046**	**0.150**
UPDRS Covariate		14	15	1.215	0.280	0.045

2. <u>Post-hoc Comparisons</u>

Table 3 – Post-hoc comparisons of turning characteristics between PD+FOG and HC, PD-FOG and HC, PD+FOG and PD-FOG. Results of the comparisons between groups in the mixed model ANOVA and mixed model ANCOVA (covariate shown in the left column). Mean difference, 95% confidence interval (CI) upper and lower bounds, and p-value are provided for each comparison. Significant effects (p < 0.05) are shown in bold. Grey shading indicates values are not applicable. Details for the uncorrected percentage in-phase comparisons are not provided as there was a significant interaction effect.

Variable	**PD+FOG - HC**				**PD-FOG - HC**				**PD+FOG - PD-FOG**			
	Mean difference	95% CI lower	95% CI upper	p-value	Mean difference	95% CI lower	95% CI upper	p-value	Mean difference	95% CI lower	95% CI upper	p-value
Percentage In-phase (%)												
MiniBEST Covariate	-1.900	-5.329	1.528	0.519	0.907	-1.973	3.788	1.000	-2.808	-5.746	0.131	0.065
UPDRS Covariate									-2.473	-5.705	0.759	0.128
Coordination Variability (°)	**4.931**	**1.330**	**8.533**	**0.005**	1.458	-2.203	5.120	0.971	**3.473**	**0.031**	**6.916**	**0.047**
MiniBEST Covariate	4.072	-0.633	8.778	0.108	0.461	-3.691	4.613	1.000	3.611	-0.327	7.550	0.081
UPDRS Covariate									2.296	-1.717	6.308	0.249

Table 4 - Post-hoc comparisons of turning characteristics between strides in the start, middle and end of the turn. Results of the comparisons between part of turn in the mixed model ANOVA and mixed model ANCOVA (covariate shown in the left column). Mean difference, 95% confidence interval (CI) upper and lower bounds, and p-value are provided for each comparison. Significant effects (p < 0.05) are shown in bold. Grey shading indicates values are not applicable. Details for the uncorrected percentage in-phase comparisons are not provided as there was a significant interaction effect.

Variable	**Start - Middle**				**Start - End**				**Middle - End**			
	Mean difference	95% CI lower	95% CI upper	p-value	Mean difference	95% CI lower	95% CI upper	p-value	Mean difference	95% CI lower	95% CI upper	p-value
Percentage In-phase (%)												
MiniBEST Covariate	**-1.220**	**-1.893**	**-0.547**	**<0.001**	**8.984**	**7.353**	**10.616**	**<0.001**	**10.204**	**8.675**	**11.734**	**<0.001**
UPDRS Covariate	-0.710	-1.762	0.343	0.289	**8.409**	**6.145**	**10.673**	**<0.001**	**9.118**	**7.038**	**11.198**	**<0.001**
Coordination Variability (°)	0.478	-1.180	2.136	1.000	**-5.742**	**-7.968**	**-3.517**	**<0.001**	**-6.220**	**-8.046**	**-4.394**	**<0.001**
MiniBEST Covariate	0.564	-1.244	2.373	1.000	**-5.307**	**-7.387**	**-3.227**	**<0.001**	**-5.872**	**-7.696**	**-4.047**	**<0.001**
UPDRS Covariate	0.711	-1.636	3.057	1.000	**-6.282**	**-9.311**	**-3.253**	**<0.001**	**-6.993**	**-9.410**	**-4.575**	**<0.001**

Table 5 - Post-hoc comparisons of turning characteristics between PD+FOG and HC, PD-FOG and HC, PD+FOG and PD-FOG. Results of the comparisons between groups in the one way ANOVA and one way ANCOVA (covariate shown in the left column). Mean difference, 95% confidence interval (CI) upper and lower bounds, and p-value are provided for each comparison. Significant effects (p < 0.05) are shown in bold.

Variable	**PD+FOG - HC**				**PD-FOG - HC**				**PD+FOG - PD-FOG**			
	Mean difference	95% CI lower	95% CI upper	p-value	Mean difference	95% CI lower	95% CI upper	p-value	Mean difference	95% CI lower	95% CI upper	p-value
Maximum angular difference (°)	6.663	-4.486	17.811	0.431	-0.688	-10.670	12.046	1.000	5.975	-5.720	17.670	0.630
MiniBEST Covariate	7.069	-9.420	23.559	1.000	1.862	-11.993	15.717	1.000	5.201	-8.925	19.339	1.000
UPDRS Covariate									2.365	-11.832	16.563	0.735
Average angular difference (°)	5.463	-2.797	13.724	0.320	-0.079	-8.337	8.494	1.000	5.384	-3.281	14.050	0.387
MiniBEST Covariate	4.828	-7.377	17.034	0.984	0.222	-10.033	10.478	1.000	4.606	-5.854	15.067	0.854
UPDRS Covariate									2.693	-7.762	13.149	0.601
Number of steps	**6.474**	**3.538**	**9.410**	**<0.001**	2.537	-0.454	5.529	0.121	**3.937**	**0.857**	**7.017**	**0.008**
MiniBEST Covariate	**6.283**	**1.955**	**10.612**	**0.002**	2.002	-1.635	5.639	0.528	**4.281**	**0.571**	**7.991**	**0.019**
UPDRS Covariate									**4.261**	**0.261**	**8.254**	**0.037**
Turn duration (s)	**3.042**	**1.301**	**4.783**	**<0.001**	1.228	-0.546	3.002	0.275	1.814	-3.641	0.012	0.052
MiniBEST Covariate	2.393	-0.084	4.870	0.061	0.541	-1.540	2.622	1.000	1.852	-0.271	3.974	0.105
UPDRS Covariate									1.145	-0.990	3.279	0.280

3. Turns Towards the Most- and Least-Affected Side

Most affected side was determined by the bilateral sub-items of the Motor Section (III) of the Movement Disorder Society-Unified Parkinson's Disease Rating Scale (UPDRS)[1] (items 3.3, 3.4, 3.5, 3.6, 3.7, 3.8, 3.15, 3.16, 3.17). Turning toward the most affected side is where the most affected side is the inside, or pivot, foot of the turn. In those with a left most affected side, counterclockwise turning would be towards the most affected side. A paired samples t-test was used to examine differences between turns towards the most- and least-affected side.

Table 6 – Turning characteristics of turning towards the most-affected side (MAS) and least-affected side (LAS) in people with PD. Results of the paired samples t-test are shown. Significant differences (p < 0.05) are shown in bold. Significant post-hoc comparisons with Bonferroni corrections are shown in superscript: (S) Significantly different to the start, (M) Significantly different to the middle, (E) Significantly different to the end

Variable	Part of turn	LAS		MAS		Interaction		Affected Side		Part of turn	
		Mean	SD	Mean	SD	F	p-value	F	p-value	F	p-value
Percentage Inphase (%)	Start^{E}	93.86	4.80	94.08	3.30	1.751	0.185	**4.483**	**0.045**	**106.034**	**<0.001**
	Middle^{E}	92.78	4.02	94.04	3.03						
	End^{S,M}	82.58	4.36	84.91	6.96						
Coordination Variability (°)	Start^{E}	7.17	3.24	8.57	5.57	0.883	0.378	0.98	0.757	**33.227**	**<0.001**
	Middle^{E}	7.58	3.33	7.29	3.77						
	End^{S,M}	13.96	7.01	13.50	6.69						

There was a lower percentage of the strides inphase when turning toward the least affected side (89.74±0.76, p=0.045) compared to turning toward the most affected side (91.01±0.69, p=0.45). The percentage of the stride inphase at the end of the turn was significantly lower (83.75±1.02) than start (93.97±0.71, p<0.001) and middle (93.41±0.65, p<0.001) of the turn. There were no differences between strides in the start and middle of the turn (p=0.493).

There were no differences between turns towards the most- and least-affected side in coordination variability. Coordination variability in strides at the end of the turn (13.73±1.19) was significantly higher than at the start (7.88±0.81, p<0.001) and middle (7.43±0.69, p<0.001). There were no differences in coordination variability between strides in the start and middle of the turn (p=1.000).

Table 7 – Turning characteristics in turns towards the most-affected side (MAS) and turns towards the least-affected side (LAS). Significant differences (p<0.05) are shown in bold.

Variable	LAS		MAS		t-test	
	Mean	SD	Mean	SD	t	p-value
Maximum angular difference (°)	24.87	13.22	23.87	15.07	0.474	0.640
Average angular difference (°)	13.43	9.00	12.33	9.07	0.550	0.587
Number of steps	13.44	4.88	13.58	4.79	0.364	0.719
Turn duration (s)	7.48	2.29	7.51	2.28	0.107	0.916

There were no significant differences in turning towards the most-affected side compared to the least affected side in maximum and average angular difference, or number of steps and Turn duration.

There were no differences between turning towards the most- and least-affected side across all variables, except for the percentage of the stride in-phase across all sections of the turn (p=0.045). Most-affected side was determined by summing all left/right components of the UPDRS. Seuthe et al.[2] found that 39% of people with PD took more time turning towards the most-affected side (determined by UPDRS), and 47% of people took more time turning towards the least affected side. There appears to be poor congruence between turning, gait and UPDRS asymmetry, with lots of inter-individual variation.

4. <u>Pearson's Correlation Analysis</u>

*Table 8 – Pearson's Correlation analysis for all participants between coordination variability at the start, middle and end of the turn and turn duration, MiniBEST and MDS-UPDRS score (with the FOG component removed). Correlations with MDS-UPDRS score could only be run in the PD+FOG and PD-FOG group. * denotes a significant correlation at the p<0.05 level. Grey shading indicates values are not applicable.*

		Coordination Variability - Start(°)	Coordination Variability - Middle(°)	Coordination Variability - End(°)	Turn duration (s)	MDS-UPDRS	MiniBEST
Coordination Variability - Start(°)	Pearson Correlation		.583*	.551*	.393*	.212	-.207
	Sig. (2-tailed)		<.001	<.001	.015	.288	.232
	N		38	38	38	27	35
Coordination Variability - Middle(°)	Pearson Correlation	.583*		.679**	.693*	.235	-.341*
	Sig. (2-tailed)	<.001		<.001	<.001	.219	.027
	N	38		46	46	29	42
Coordination Variability - End(°)	Pearson Correlation	.551*	.679*		.387*	.196	-.406*
	Sig. (2-tailed)	<.001	<.001		.008	.307	.008
	N	38	46		46	29	42
Turn duration (s)	Pearson Correlation	.393*	.693*	.387*		.390*	-.445*
	Sig. (2-tailed)	.015	<.001	.008		.036	.003
	N	38	46	46		29	42
MDS-UPDRS	Pearson Correlation	.212	.235	.196	.390*		-.335
	Sig. (2-tailed)	.288	.219	.307	.036		.087
	N	27	29	29	29		27
MiniBEST	Pearson Correlation	-.207	-.341*	-.406*	-.445*	-.335	
	Sig. (2-tailed)	.232	.027	.008	.003	.087	
	N	35	42	42	42	27	

5. Statistical Parametric Mapping (SPM) Results

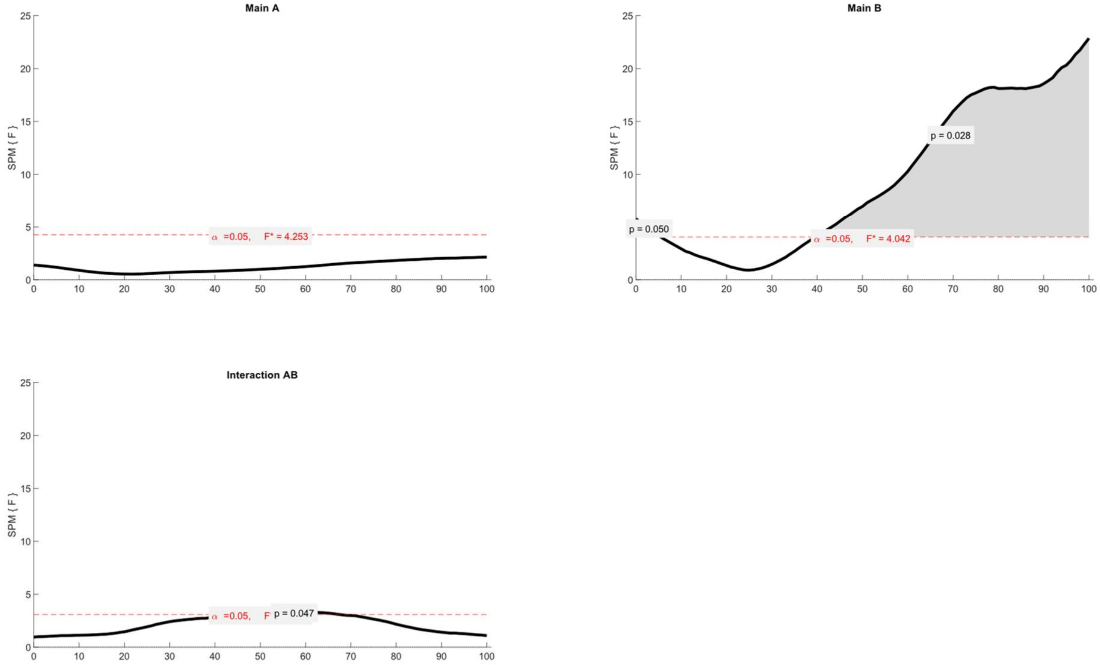

Figure 1 - Results from SPM two-way ANOVA with repeated-measures on one factor comparing angular difference across the stride between start, middle and end of the turn, and PD+FOG, PD-FOG and HC. Main A refers to the main effect of group, Main B to the main effect of part of turn, and Interaction AB to the interaction between group and part of turn. Non-sphericity corrections have not been applied within the SPM1D package, so caution is recommended when interpreting values close to the alpha. For this reason, the very slight amount of the stride above the threshold of the Interaction AB has been disregarded.

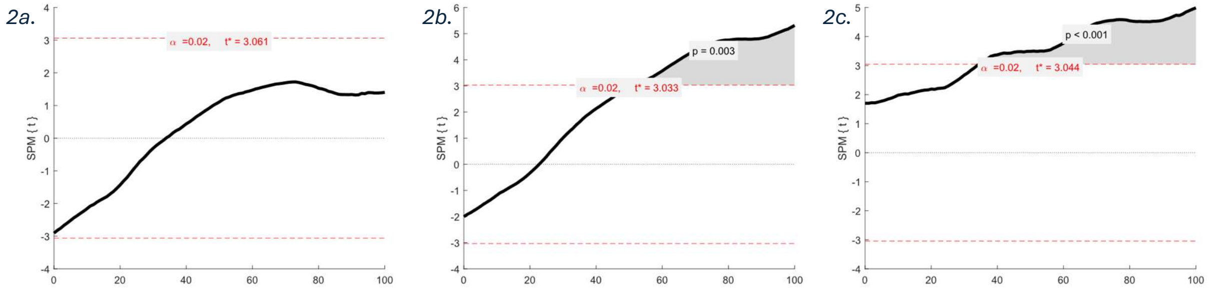

Figure 2 - Comparisons of the main effect of part of turn on angular difference with Bonferroni post-hoc corrections applied. 2a. represents comparison between strides in the start of the turn and the middle; 2b. represents comparison of strides in the start of the turn to the end; 2c. represents comparison between strides in the middle of the turn to the end.

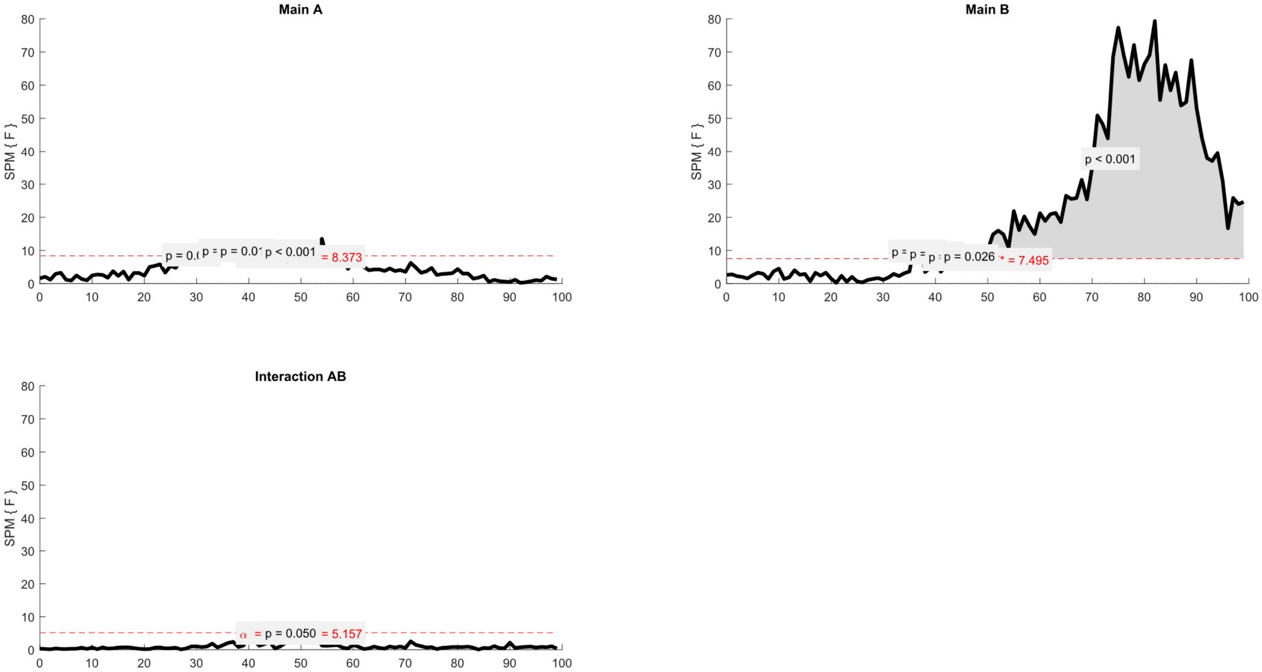

Figure 1 – Results from SPM two-way ANOVA with repeated-measures on one factor comparing coordination variability across the stride between start, middle and end of the turn, and PD+FOG, PD-FOG and HC. Main A refers to the main effect of group, Main B to the main effect of part of turn, and Interaction AB to the interaction between group and part of turn. Non-sphericity corrections have not been applied within the SPM1D package, so caution is recommended when interpreting values close to the alpha. For this reason, the very slight amount of the stride above the threshold of the Interaction AB has been disregarded.

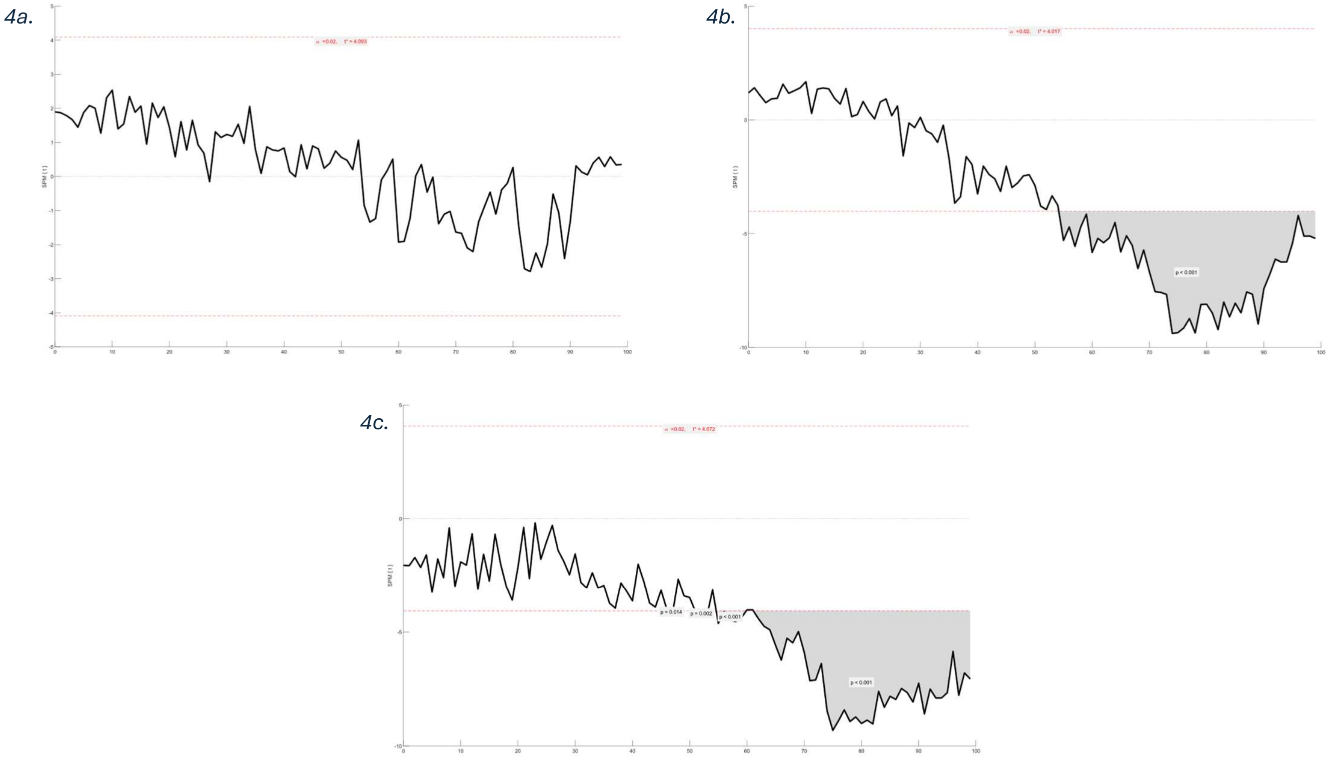

Figure 2 - Comparisons of the main effect of part of turn on coordination variability with Bonferroni post-hoc corrections applied. 4a. represents comparison between strides in the start of the turn and the middle; 4b. represents comparison of strides in the start of the turn to the end; 4c. represents comparison between strides in the middle of the turn to the end.

5a.

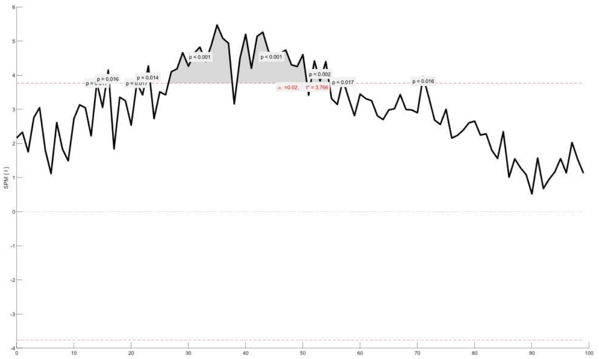

5b.

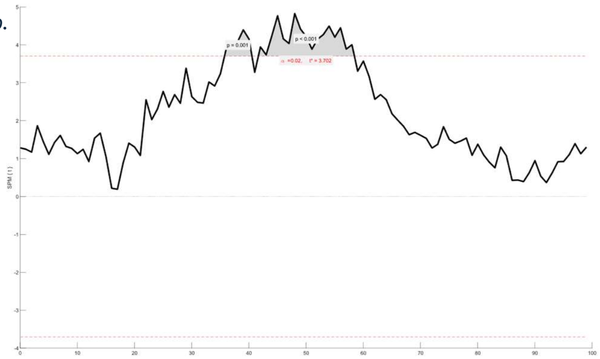

5c.

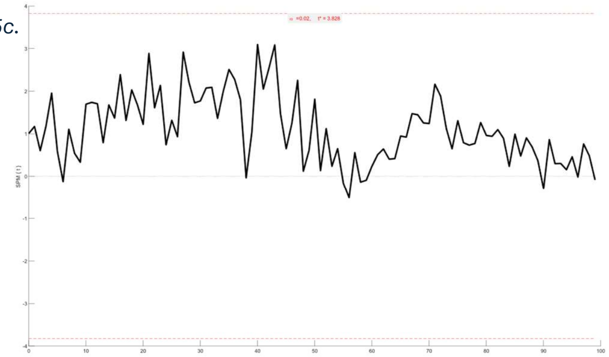

Figure 5 - Comparisons of the main effect of group on coordination variability with Bonferroni post-hoc corrections applied. 5a. represents comparison between strides in PD+FOG and HC; 5b. represents comparison of strides in PD+FOG and PD-FOG; 5c. represents comparison between PD-FOG and HC.

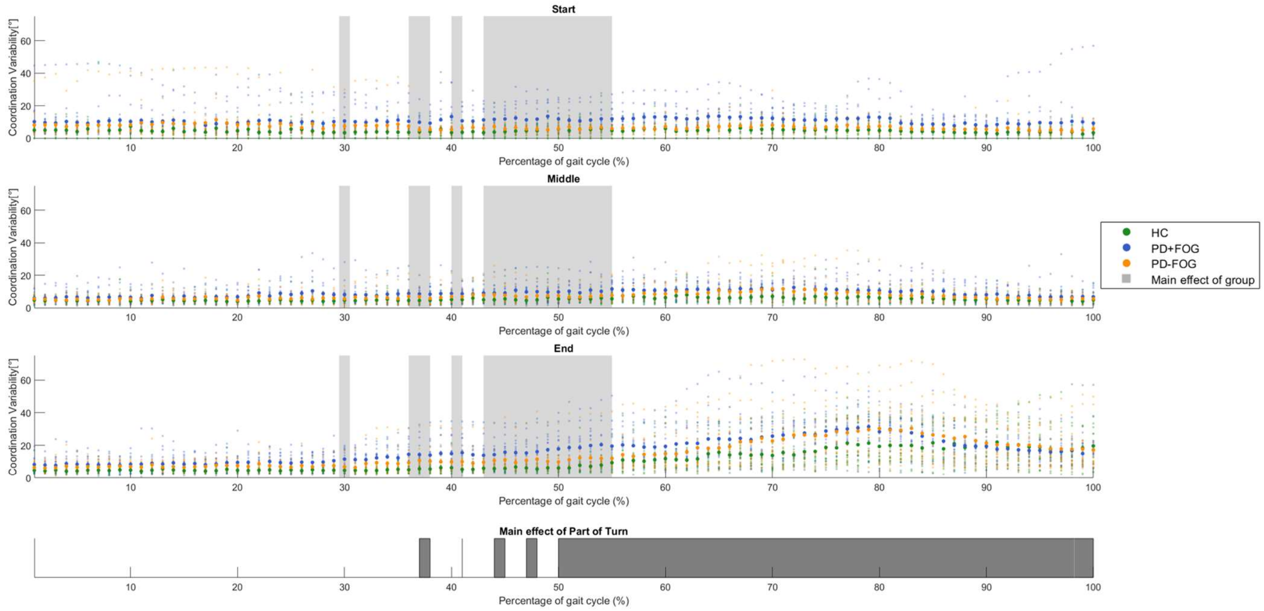

Figure 6 - Head-pelvis coordination variability across the gait cycle at the start, middle and end of the turn in HC (green), PD+FOG (blue) and PD-FOG (orange) groups. Transparent data points represent coordination variability for each participant, and bold points represent the mean for each group. Significant main effect of group is highlighted in light grey on each graph. Significant main effects of part of turn are presented in the bottom plot.

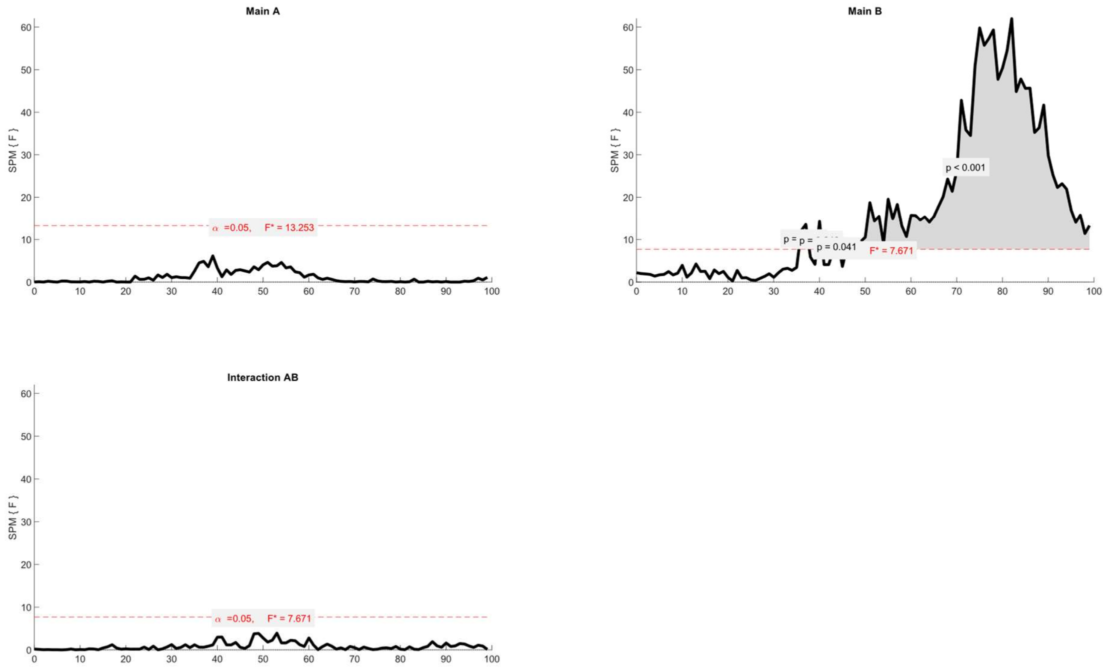

Figure 7 - SPM regression between coordination variability across the stride and UPDRS score was run and a two-way ANOVA with repeated-measures on one factor used to compare residuals between start, middle and end of the turn, and PD+FOG, PD-FOG and HC. Main A refers to the main effect of group, Main B to the main effect of part of turn, and Interaction AB to the interaction between group and part of turn.

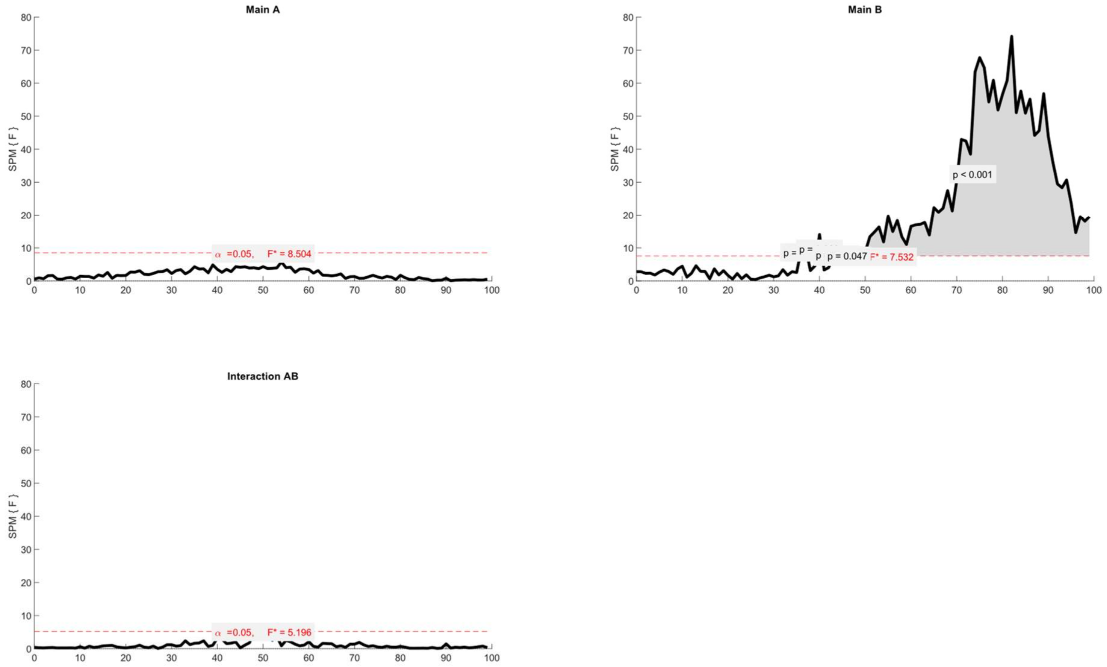

Figure 8 - SPM regression between coordination variability across the stride and miniBEST score was run and a two-way ANOVA with repeated-measures on one factor used to compare residuals between start, middle and end of the turn, and PD+FOG, PD-FOG and HC. Main A refers to the main effect of group, Main B to the main effect of part of turn, and Interaction AB to the interaction between group and part of turn.

6. Gait event identification

Heel strikes were semi-automatically labelled by calculating the cumulative arc length of the heel marker trajectory. Only heel strike was required for this analysis. Local maxima of the cumulative Arc Length gave index of heel strike.

Heel marker trajectory is given as a list of x, y, and z coordinates, where $P_i = [x_i, y_{i,} z_i]$. The cumulative arc length of the heel marker trajectory is computed as the sum of the Euclidean distances between consecutive points along the time series. The arc length at the i-th point is defined as:

$$L_{(i)} = L_{(i-1)} + \| P_i - P_{i-1} \|$$

Where:

$$L_1 = 0$$

Here, $\| P_i - P_{i-1} \|$ represents the Euclidean distance between consecutive points P_i and P_{i-1} given by:

$$\| P_i - P_{i-1} \| = \sqrt{(x_i - x_{i-1})^2 + (y_i - y_{i-1})^2 + (z_i - z_{i-1})^2}$$

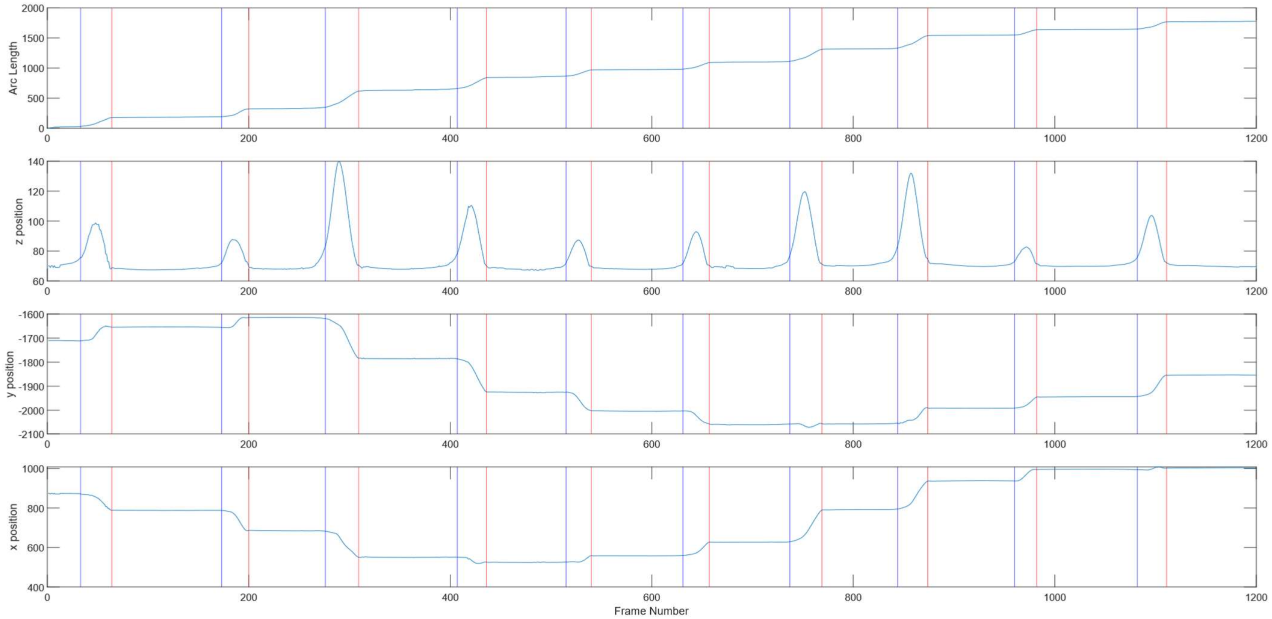

Figure 3 – Example of (right) heel marker trajectory and arc length in PD+FOG participant. Vertical red lines show heel strike, vertical blue lines show heel off based on local maxima and minima of the arc length, respectively.

7. Inclusion/Exclusion for each Participant

Table 9 – UPDRS and miniBEST scores are presented for each participant included in the analysis. Participants whose data were collected but excluded are indicated by a fully greyed-out row, with the reason for exclusion specified. Inclusion to Analysis columns corresponds to different comparisons, with one column dedicated to each analysis. Coordination variability and angular difference comparisons are combined into a single column, as all participants included in the discrete analysis were also included in the continuous SPM analysis. Greyed-out cells indicate participants excluded from specific analyses, with the reason for exclusion provided. The number of turns analysed for each participant is reported, and the total number of participants included in each analysis (n) is given at the end of each group.

		Scores					Inclusion to Analysis							
Group	Participant ID	UPDRS Total	UPDRS Left	UPDRS Right	UPDRS FOG score	miniBEST score	MAS/LAS	Inphase %	Angular difference	Coordination Variability	Number of steps	Turn time	Number of turns each direction	Demographic data
PD+FOG	FOG1	Excluded due to technical issues with data												
	FOG2	Testing never completed												
	FOG3	MOCA Score <20												
	FOG4	Excluded due to comorbidities that would affect turning												
	FOG5	Excluded due to technical issues with data												
	FOG6	49	21	10	1	21							5 CCW, 5 CW	
	FOG7	40	12	11	1	24							3 CCW, 5 CW	
	FOG8	45	14	10	2	11							8 CCW, 5 CW	
	FOG9	36	11	12	2	15							5 CCW, 5 CW	
	FOG10	43	10	14	2	12							5 CCW, 5 CW	
	FOG11	Insufficient 360° turns for analysis												
	FOG12	Excluded due to technical issues with data												
	FOG13	39	14	10	2	8							5 CCW, 5 CW	

	FOG14	29	12	6	0	18							5 CCW, 5 CW	
	FOG15	57	17	18	3	23							5 CCW, 5 CW	
	FOG16	36	10	6	2	23							5 CCW, 5 CW	
	FOG17	63	21	21	2	10	No most-affected side			One or No strides in first third of turn			5 CCW, 5 CW	
	FOG18	47	17	12	2	21							5 CCW, 5 CW	
	FOG19	28	13	7	0	17							5 CCW, 5 CW	
	FOG20	34	11	9	1	19							5 CCW, 5 CW	
	FOG21	Insufficient complete 360° turns for analysis												
	FOG22	37	11	7		19							5 CCW, 5 CW	
	FOG23	34	12	11		25							5 CCW, 5 CW	
	Total						14	15	15	14	15	15		
	NFOG1	21	8	8		27	No most-affected side						5 CCW, 5 CW	
PD-FOG	NFOG2	31	18	8	No miniBEST score								5 CCW, 5 CW	
	NFOG3	Excluded due to technical issues with data												

NFOG4	20	7	4		17			5 CCW, 5 CW	
NFOG5	31	12	16		28			5 CCW, 5 CW	
NFOG6	24	6	7		22			5 CCW, 5 CW	
NFOG7	45	9	14		20			5 CCW, 5 CW	
NFOG8	25	12	8		22		One or No strides in first third of turn	5 CCW, 5 CW	
NFOG9	16	7	2		22			5 CCW, 5 CW	
NFOG10	35	8	15		19			5 CCW, 5 CW	
NFOG11	13	5	0		20			5 CCW, 5 CW	
NFOG12	10	1	0		17			5 CCW, 5 CW	
NFOG13	15	1	9		27			5 CCW, 5 CW	
NFOG14	33	9	9		No miniBEST score	No most-affected side		5 CCW, 5 CW	Missing H&Y score
NFOG15	Excluded due to technical issues with data								
NFOG16	Excluded due to technical issues with data								
NFOG17	Did not complete the protocol								
NFOG18	Excluded due to technical issues with data								

	NFOG19	30	12	11	0	27							5 CCW, 5 CW	
	Total						12	14	14	13	14	14		
	HC1					27				One or No strides in first third of turn			5 CCW, 5 CW	
	HC2								Missing data					
	HC3					No miniBEST score				One or No strides in first third of turn			5 CCW, 5 CW	
	HC4					30				One or No strides in first third of turn			5 CCW, 5 CW	
HC	HC5								Excluded due to technical issues with data					
	HC6								Excluded due to technical issues with data					
	HC7								Excluded due to technical issues with data					
	HC8								Excluded due to technical issues with data					
	HC9								Excluded due to technical issues with data					
	HC10					` No miniBEST score							5 CCW, 5 CW	
	HC11					28				One or No strides in first third of turn			5 CCW, 5 CW	
	HC12					25							5 CCW, 5 CW	

HC13		25				One or No strides in first third of turn			5 CCW, 5 CW	
HC14		26							5 CCW, 5 CW	
HC15		Excluded due to technical issues with data								
HC16		23							5 CCW, 5 CW	
HC17		28							5 CCW, 5 CW	
HC18		28							5 CCW, 5 CW	
HC19		27				One or No strides in first third of turn			5 CCW, 5 CW	
HC20		26							5 CCW, 5 CW	
HC21		25							5 CCW, 5 CW	No age recorded
HC22		28							5 CCW, 5 CW	
HC23		22							5 CCW, 5 CW	
HC24		28							5 CCW, 5 CW	
Total				17	17	11	17	17		

8. <u>References</u>

1. Goetz, C. G. *et al*. Movement Disorder Society-sponsored revision of the Unified Parkinson's Disease Rating Scale (MDS-UPDRS): Scale presentation and clinimetric testing results. *Movement Disorders* **23**, 2129–2170 (2008).
2. Seuthe, J. *et al*. Gait asymmetry and symptom laterality in Parkinson's disease: two of a kind? *J Neurol* **271**, 4373–4382 (2024).
